# Heterogeneity in semen-mediated tolerogenic responses: defining immunological determinants of reproductive compatibility

**DOI:** 10.1093/hropen/hoag064

**Published:** 2026-07-15

**Authors:** Shahrokh Paktinat, Michael G Gravett, Sean M Hughes, Abigail L P Spray, Germán G Gornalusse, Florian Hladik, Lucia Vojtech

**Affiliations:** Department of Obstetrics and Gynecology, University of Washington, Seattle, WA, USA; Department of Obstetrics and Gynecology, University of Washington, Seattle, WA, USA; Department of Obstetrics and Gynecology, University of Washington, Seattle, WA, USA; Department of Obstetrics and Gynecology, University of Washington, Seattle, WA, USA; Department of Obstetrics and Gynecology, University of Washington, Seattle, WA, USA; Vaccine and Infectious Disease Division, Fred Hutchinson Cancer Center, Seattle, WA, USA; Department of Obstetrics and Gynecology, University of Washington, Seattle, WA, USA; Vaccine and Infectious Disease Division, Fred Hutchinson Cancer Center, Seattle, WA, USA; Department of Medicine, University of Washington, Seattle, WA, USA; Department of Obstetrics and Gynecology, University of Washington, Seattle, WA, USA

**Keywords:** semen, extracellular vesicle, immunity, immune tolerance, regulatory T cell, dendritic cell

## Abstract

**STUDY QUESTION:**

Do particular pairs of individuals modeling sexual pairs exhibit differences in the induction of semen-mediated long-lasting tolerance in support of pregnancy?

**SUMMARY ANSWER:**

Yes, factors from both sexes contribute to the variability in the induction of long-lived regulatory T cells (Tregs) in response to semen, with factors related to the recipient seeming to slightly outweigh the factors related to the semen donor.

**WHAT IS KNOWN ALREADY:**

Semen contains multiple immunomodulatory factors that can influence reproductive tract immunity in the recipient, promote tolerance to antigens from the semen donor (designated henceforth as ‘paternal’), and ultimately support successful pregnancy. These factors include the soluble fraction containing prostaglandins and cytokines, as well as a very high concentration of extracellular vesicles (EVs). Both soluble and vesicular fractions of semen have been shown to promote tolerogenic phenotypes and migration in antigen-presenting cells (APCs), and to induce Tregs. This study was undertaken to understand the variation in tolerance induction between pairs of individuals modeling sexually active couples.

**STUDY DESIGN, SIZE, DURATION:**

We designed an *in vitro* pairing study using random semen donors (N = 11) and peripheral mononuclear cells (PBMCs) from random self-identified female recipients (N = 3). Semen was fractionated into vesicle-enriched (semen extracellular vesicles; SEVs) and soluble (vesicle-depleted semen plasma; VDSP) fractions. Monocyte-derived dendritic cells (MoDCs) from each of the recipients were exposed to semen fractions from each donor, then cocultured with autologous naïve T cells to assess tolerogenic responses, specifically, generation of Tregs (FOXP3+ CD25Hi CD127Lo CD4+ T cells) and their phenotypic differences (TIGIT+, PD-1+ CTLA-4+, or AREG+ Tregs). These cells were then screened for T cell receptor-dependent activation by activation-induced marker (AIM) expression assay. These responses were compared across all pairs of semen donors and recipient cells. In addition, semen fractions were screened for candidate mediators of tolerance, such as human leukocyte antigen G (HLA-G) and adenosine-generating enzymes.

**PARTICIPANTS/MATERIALS, SETTING, METHODS:**

This study used an *in vitro* human cell culture approach in which primary blood-derived immune cells were exposed to semen-derived vesicular and non-vesicular fractions. Blood samples were obtained from healthy self-identified female donors, and semen samples were obtained from healthy participants who provided initial screening specimens for a contraceptive study. Semen-derived vesicular and non-vesicular fractions were characterized by western-blot analysis for markers associated with immune tolerance. Primary immune cells were exposed to these semen fractions, and the major endpoints included changes in Treg phenotypes and subset frequencies, as well as AIM expression. These outcomes were assessed by flow cytometry. No animal models were used.

**MAIN RESULTS AND THE ROLE OF CHANCE:**

HLA-G content in semen (SEV and VDSP) varied markedly across donors, ranging from undetectable to high concentrations. Similarly, semen’s capacity to generate adenosine from ATP differed by ∼4-fold among donors. Induction of Tregs by SEV and VDSP from 11 semen donors was highly variable, from 2.8% to 63.4% of CD4+ T cell population. Semen fractions from a few donors consistently elicited lower tolerogenic responses across all recipients. However, when we considered the combined effects of both semen fractions, none of the semen donors failed to elicit a measurable tolerogenic response. On the other hand, one recipient was a consistently low responder to tolerance induction. Thus, both recipient immune cells’ features and semen-intrinsic properties are significant determinants of tolerogenic responsiveness. Semen re-exposure enhanced Treg AIM expression. The strongest effect was observed when Tregs were exposed to VDSP-loaded MoDCs. In contrast, SEV-loaded MoDCs caused a much weaker response.

**LARGE SCALE DATA:**

N/A

**LIMITATIONS, REASONS FOR CAUTION:**

One limitation of our study is that we used semen and circulating recipient cells from random donors not selected for any clinical characteristics, such as history of infertility. In addition, we did not correlate HLA-G content and adenosine-generating enzyme expression levels with the Treg and AIM readouts. We show, via Treg activation, that semen induces tolerance to its antigens, but we do not demonstrate which specific antigens are recognized. We posit that the variation in semen-induced tolerance observed between couples plays a role in reproductive outcomes; however, larger studies in clinically characterized cohorts and using cells from the tissue microenvironment will be required to establish this link.

**WIDER IMPLICATIONS OF THE FINDINGS:**

Our findings demonstrate that semen-induced tolerance is highly variable and may be more strongly shaped by recipient factors than by semen donor factors. In addition, the generation of Tregs with enhanced activation to previously encountered semen supports the concepts of trained immunity and antigen-specific tolerance that arise cumulatively following exposure to semen from the partner. These Tregs are poised to expand and support pregnancies with fetuses expressing paternal alloantigens.

**FUNDING:**

This work was supported by the following grants: National Institutes of Health/Eunice Kennedy Shriver National Institute of Child Health and Human Development (R01AI153342) to L.V., National Institutes of Health/National Institute on Drug Abuse (R01DA040386) to F.H., National Institutes of Health/National Center for Advancing Translational Sciences (KL2TR002317) to G.G.G.

**DISCLOSURES:**

The authors have no conflicts of interest to declare.

WHAT DOES THIS MEAN FOR PATIENTS? It is very important for the immune system to tolerate the fetus during pregnancy. Previous studies have shown that exposure to semen helps initiate this tolerance. In this study, we tested combinations of random unrelated semen samples and recipient immune cells to determine how much variation there is between experimental couples in this tolerance response. We used a protein detection technique to look at variation in semen molecules that promote tolerance. We used a cell detection technique to look at differences in recipient immune cells treated with semen from different donors. We found that more of the variation in tolerance was due to the recipient immune cells, and less due to the individual semen donor. We think some cases of fertility issues or pregnancy diseases, such as recurrent pregnancy loss and preeclampsia, could be due to low tolerance induction in particular couples. In the future, measuring tolerance to semen might be a way to diagnose otherwise unknown causes of infertility.

## Introduction

The involvement of the immune system in pregnancy has emerged as a central topic in reproductive biology. It is now well established that pregnancy is characterized by dynamic immune adaptation with immune cells providing critical regulatory and trophic signals that support decidual and placental development (Hosking *et al.*, 2024; Li *et al.*, 2024). A deeper understanding of these immune processes is essential for elucidating the pathophysiology of pregnancy complications and for developing innovative therapeutics for infertility.

Adaptive immune tolerance to the semi-allogeneic fetus is a central determinant of a healthy pregnancy. Simultaneously, the capacity to mount effective immune responses against pathogens must be preserved (Alippe *et al.*, 2025; Li *et al.*, 2025). Epidemiological and clinical evidence suggests that exposure to semen pre-conception initiates the immune environment optimal for pregnancy health. Preeclampsia (PE) is a disease linked to disrupted immune adaptation that occurs primarily in first pregnancies. Risk of PE is greater following the use of donor sperm in ART, and with reduced prior exposure to semen via shorter duration of sexual relationships or use of condoms for contraception (Smith *et al.*, 1997; Kho *et al.*, 2009; Kyrou *et al.*, 2010; Saftlas *et al.*, 2014; Hendin *et al.*, 2023). Similarly, in mouse studies, the absence of semen plasma at conception can adversely affect offspring health, and semen plasma exposure before embryo transfer can partly mitigate these effects (Bromfield *et al.*, 2014).

The noncellular compartment of semen contains many bioactive factors including inflammatory/regulatory cytokines such as TGF-β isoforms and IL-10 (Huleihel *et al.*, 1999; Loras *et al.*, 1999; Politch *et al.*, 2007; Sharkey *et al.*, 2016); chemokines such as CXCL8 (IL-8) (Politch *et al.*, 2007; Sharkey *et al.*, 2017); growth factors such as GM-CSF (Politch *et al.*, 2007; Placzkowska *et al.*, 2024) and VEGF (Obermair *et al.*, 1999), and lipid mediators and hormones, including prostaglandins (Templeton *et al.*, 1978) and steroids (Olesti *et al.*, 2020). Semen also contains variable amounts of membrane-bound and soluble isoforms of human leukocyte antigen G (HLA-G) (Larsen *et al.*, 2011; Schallmoser *et al.*, 2019), a nonpolymorphic HLA with well-known immunosuppressive effects including inhibition of NK cell-mediated cell lysis (Riteau *et al.*, 2001) and inhibition of allogeneic T-cell proliferation while promoting regulatory T-cell (Treg) expansion (Selmani *et al.*, 2008). Biopsies or cervicovaginal lavage obtained after coitus show elevated IL-6 and monocyte chemotactic protein (MCP)-1 (Francis *et al.*, 2016), and a marked influx of leukocytes, including CD14+ macrophages and CD1a+ dendritic cells (Sharkey *et al.*, 2012). These observations have also been examined *in vitro* using immortalized and primary cervical and vaginal epithelial cells, which respond to semen plasma by secreting GM-CSF, IL-6, IL-8, and MCP-1 (Sharkey *et al.*, 2007). Notably, levels of immune modulators including TGF-β vary in semen over time (Sharkey *et al.*, 2016), and exposure to a panel of semen plasma samples from healthy proven-fertile donors revealed substantial interindividual variation in this cytokine-inducing activity (Sharkey *et al.*, 2007).

Semen also has a high concentration of extracellular vesicles (EVs) capable of directly inducing T cells (Zhang *et al.*, 2024), or of driving tolerogenic programs in antigen-presenting cells (APCs) which promote long-lived Tregs (Robertson *et al.*, 2009, 2013; Robertson and Sharkey, 2016). Because dendritic cells are abundant in cervical and vaginal mucosa and are specialized for antigen capture and T-cell priming (Joo *et al.*, 2025), they are well positioned to mediate how semen shapes immune activation and regulation. In our previous work (Paktinat *et al.*, 2025), we showed that semen extracellular vesicles (SEVs) drive the migration of vaginal mucosal APCs out of tissue. These migrating tolerogenic APCs could migrate to lymph nodes to deliver signals to naïve T cells, thereby promoting their differentiation into Tregs poised to expand upon conception to support pregnancy (Shima *et al.*, 2015, 2020). We demonstrated this effect for both SEV and vesicle-depleted semen plasma (VDSP), highlighting that both fractions may contribute to the immunological functions of semen in reproduction. However, the relative contributions of semen donors versus recipients to the observed variability at the couple level remain undefined.

Altogether, these findings are compatible with a coordinated cumulative adaptive response to semen which may differ between sexual partners. Failure of this response in particular couples may contribute to reproductive disorders, but whether semen or recipient responses play a larger role remains unknown. To address these gaps, we established a framework to test whether variability in reproductive outcomes may reflect partner-specific differences in tolerance induction. Specifically, we asked whether semen from a given donor elicits consistently weak tolerogenic responses across recipients or whether responses were recipient-dependent. We also asked whether some recipients mount broadly robust tolerogenic programs or instead respond selectively to particular semen samples. We first examined candidate mediators of semen’s immunological activity, including HLA-G and additional factors likely to contribute to semen’s immunomodulatory functions. Then, we applied an *in vitro* pairing design in which semen samples were paired with immune cells from multiple recipients. This approach enabled quantification of Treg induction and phenotypic shifts while separating semen donor effects, immune-cell donor (semen recipient) effects, and donor-recipient interactions that may contribute to variability in tolerance-associated responses. Two patterns emerged: variability in recipient responsiveness, spanning low to high responders, and variability in semen potency, with a subset of semen samples eliciting robust tolerogenic effects across multiple recipients. Notably, recipient-associated factors appeared to contribute more strongly to this variability than semen donor-associated factors. Together, these findings support a model in which both semen- and recipient-intrinsic properties shape tolerogenic responses within donor–recipient pairings, providing a rationale for larger studies that relate these immunological phenotypes to clinical outcomes in couples with healthy versus adverse pregnancies. Ultimately, such work could inform the development of clinically useful approaches to assess immunological compatibility in human couples.

## Materials and methods

### Human subjects

Semen specimens were obtained from normal volunteers without a history of vasectomy, testicular trauma, or infertility who were recruited for contraceptive studies in Dr. John Amory’s laboratory at the University of Washington Medical Center under IRB-approved protocols and stored at −20°C until fractionation. All participants provided written informed consents before semen samples were obtained. Prior to freezing, all samples underwent analysis for sperm concentration, count, motility, and morphology using WHO methods in Dr. Amory’s lab. Samples were also analyzed for the presence of round cells and agglutination, which could indicate inflammation. Peripheral blood samples were obtained from healthy self-identified female donors at Bloodworks Bioresearch program (Bloodworks Northwest, Seattle, WA, USA). Parity and pregnancy history were not collected. Peripheral blood mononuclear cells (PBMCs) were immediately isolated using Lymphoprep™ (STEMCELL Technologies, Vancouver, BC, Canada) and SepMate™ (STEMCELL Technologies) tubes according to the manufacturer’s instructions and cryopreserved for subsequent use.

### Fractionation of semen

Semen samples were thawed and fractionated according to our published protocol (Vojtech *et al.*, 2014). Briefly, samples were centrifuged at 2000*g* to pellet and discard cellular components. The resulting supernatant was filtered through a 0.45 µm syringe filter to remove residual debris. EVs were isolated using two-step sucrose gradient ultracentrifugation. In the first step, the supernatant was layered over a 30% sucrose cushion prepared in deuterium oxide (D_2_O) and ultracentrifuged for 90 min at 100 000*g* using an ultracentrifuge (Beckman Coulter, Brea, CA, USA). The resulting supernatant was then subjected to a second ultracentrifugation over a 25% sucrose cushion for 10 h at the same speed. Sucrose cushions from both steps were combined and diluted in 10 ml Dulbecco’s phosphate-buffered saline (DPBS) for washing using Amicon 100 kDa centrifugal filters (MilliporeSigma, Burlington, MA, USA). The remaining supernatant from the second ultracentrifugation step was aliquoted and stored at −80°C as the VDSP fraction. The isolated SEVs were also aliquoted and stored at −80°C. The size distribution of SEV was characterized using nanoparticle tracking analysis (NTA; NanoSight NS300, Malvern Panalytical, Malvern, UK). In selected experiments for western blotting, SEV underwent further purification by size-exclusion chromatography (SEC) using qEVoriginal 70 nm columns (Izon Science Limited, Christchurch, New Zealand).

### Western-blot analysis

To assess the immunomodulatory molecules across semen fractions, western blotting was performed on SEV preparations and VDSP. Protein extracts were prepared in NP-40 lysis buffer (Boston Bioproducts, Milford, MA, USA) containing protease inhibitors (sc-24948, Santa Cruz Biotechnology, Dallas, TX, USA), quantified by BCA assay (Pierce BCA Protein Assay, Thermo Fisher Scientific, Waltham, MA, USA), and denatured at 70°C for 10 min in LDS loading buffer (NP007, NuPAGE, Invitrogen, Thermo Fisher Scientific) with 2-mercaptoethanol (M-3148, Sigma-Aldrich, St. Louis, MO, USA). Approximately 30 μg of protein per sample was separated by SDS–PAGE (Bolt™ Bis-Tris Plus Mini Gels, 4–12%, Thermo Fisher Scientific) and transferred to PVDF membranes (LC2002, Life Technologies, Thermo Fisher Scientific) in the presence of antioxidant (BT0005, Bolt Antioxidant, Thermo Fisher Scientific) in both SDS–PAGE and transfer steps. Membranes were blocked in Tris-buffered saline with 0.1% Tween 20 detergent (TBS-T) containing 5% non-fat dry milk, incubated overnight at 4°C with primary antibodies against HLA-ABC (14-9983-82, eBioscience, Thermo Fisher Scientific), HLA-C (ab126722, Abcam, Cambridge, UK) HLA-DR (sc-69673, Santa Cruz Biotechnology), HLA-DR, DP, DQ (555557, BD Biosciences, San Jose, CA, USA), HLA-E (MCA2193, Bio-Rad, Hercules, CA, USA), HLA-G (557577, BD Biosciences), CD39 (NBP2-25223SS, Novus Biologicals, Centennial, CO, USA), and CD73 (13160T, Cell Signaling Technology, Danvers, MA, USA) followed by HRP-conjugated secondary antibodies (anti-mouse, 31430, Thermo Fisher Scientific; or anti-rabbit, EXOAB-HRP, System Biosciences, Newark, CA, USA). Blots were developed using SuperSignal™ West Pico PLUS Chemiluminescent Substrate (Thermo Fisher Scientific) and imaged on a ChemiDoc MP Imaging System (Bio-Rad).

### Adenosine quantification assay

To assess CD73 enzymatic activity, SEV and VDSP fractions were incubated with 1 mM ATP (U0455, Abnova, Taipei, Taiwan) at 37°C for 30 min to allow substrate hydrolysis. Following incubation, adenosine, the end metabolite of CD73-mediated dephosphorylation, was quantified using a fluorometric assay (MAK433, Adenosine Quantification Kit, Sigma-Aldrich) according to the manufacturer’s protocol.

### Generation and stimulation of monocyte-derived dendritic cells

To evaluate the immunological effects of semen fractions on recipient cells, we used monocyte-derived dendritic cells (MoDCs) as APCs. Cryopreserved PBMC were thawed and monocytes were immediately isolated using a negative selection kit (19359, STEMCELL Technologies), according to the manufacturer’s instructions. Purified monocytes were differentiated into dendritic cells as described previously (Vojtech *et al.*, 2019), with minor modification. Briefly, monocytes we cultured in ImmunoCult™ medium (10987, STEMCELL Technologies) supplemented with recombinant human GM-CSF (800 IU/ml) and IL-4 (250 IU/ml; PeproTech, 300-03 and 200-04, respectively, Thermo Fisher Scientific) for 5 days, with a media change on Day 3. After 5 days, the cells were collected: non-adherent cells were harvested by gentle pipetting, and adherent cells were detached using DPBS containing 2.5 mM EDTA after a 20-min incubation. The combined cell suspension was washed, counted, and assessed for viability using Muse Count & Viability Reagent on a Muse Cell Analyzer (Luminex/Guava Technologies, Cytek Biosciences, Fremont, CA, USA). MoDCs were then incubated overnight with either SEV or VDSP both at a fixed 10% (v/v) concentration in 100 µl of AIM V™ medium (Gibco, Grand Island, NY, USA) in 96-well plates (15 000–20 000 MoDCs per well).

### Coculture of semen-loaded MoDCs with naïve CD4+ T cells

To evaluate the functional capacity of semen-exposed MoDCs to induce Treg differentiation, naïve CD4+ T cells were isolated from peripheral blood using a negative selection kit (17555, STEMCELL Technologies) and cocultured with semen-loaded (i.e. SEV- or VDSP-exposed) autologous MoDCs at a 1:3 ratio (MoDC: T cell) for 7 days, as previously described (Paktinat *et al.*, 2025). Cultures were maintained in AIM V™ medium supplemented with recombinant human interleukin-2 (200 IU/ml; PeproTech, 200-02, Thermo Fisher Scientific). At the end of the coculture period, cells were stained and analyzed for the expression of Treg lineage and immunoregulatory markers by flow cytometry (Paktinat *et al.*, 2025).

### Experimental setup for activation-induced marker assay

To evaluate antigen responsiveness of *in vitro*-differentiated Tregs, we restimulated them with autologous MoDCs freshly loaded with the original semen fraction and incubated overnight. Cells were then assessed for surface expression of activation-induced markers (AIMs), which is widely used as a readout for T cell receptor (TCR)-dependent activation (Zheng *et al.*, 2025). To assess the specificity of this response to a given semen sample, we included a comparator condition in which the cells were restimulated with MoDCs loaded with a semen fraction from a different donor.

### Flow cytometry staining and data acquisition

Flow cytometry panels used for staining regulatory CD4+ T cells and for the AIM assay are summarized in Supplementary Tables S1 and S2. Briefly, cells were washed with DPBS and stained with a viability dye in combination with an Fc receptor-blocking reagent (Human TruStain FcX™, BioLegend, San Diego, CA, USA). Surface staining was performed using a cocktail of fluorochrome-conjugated antibodies. Cells were then fixed and permeabilized (eBioscience™ Foxp3/Transcription Factor Fixation/Permeabilization kit, Thermo Fisher Scientific) for intracellular and intranuclear staining, followed by final fixation in 4% paraformaldehyde (PFA). Data were acquired on a Cytek Aurora 5-laser spectral flow cytometer (Cytek Biosciences). Unstained and single-stained reference controls were used for unmixing.

### Data analysis and visualization

Flow cytometry data were analyzed using FlowJo™ software (version 10, BD Biosciences). Data visualization was performed using GraphPad Prism (version 10, GraphPad Prism, Boston, MA, USA). Heatmaps were selected as the primary visualization method to facilitate integrated comparisons across all conditions. Experiments were performed on separate days and in duplicate to minimize potential bias introduced by day-to-day or well-to-well variabilities.

## Results

### Immune-regulatory molecules detected in semen compartments

#### Rationale and experimental design

To investigate the immune functions of semen, we focused on two classes of molecules with potential immunoregulatory roles: (i) major histocompatibility complex (MHC) molecules, such as HLA-G, which are implicated in tolerance to the semi-allogeneic fetus (Xu *et al.*, 2020; Aisagbonhi and Morris, 2022), and (ii) the ectonucleotidases, CD39 and CD73, which contribute to immune suppression through the generation of extracellular adenosine (Clayton *et al.*, 2011). Although the presence of HLA-G in semen has been reported, differences in its distribution between semen compartments have not been examined. Similarly, the presence, localization, and functional activity of CD39 and CD73 within these fractions remain less characterized, with only one report documenting the presence of CD73 (known as 5′-Nucleotidase Ecto; NT5E) in the SEV fraction (Zhang *et al.*, 2020).

To address these gaps, we used western blotting to assess MHC molecules and these two ectonucleotidases in SEV and VDSP fractions. In select experiments, SEV preparations underwent additional purification to obtain highly purified SEV isolates. Additionally, we conducted a functional assay to assess the enzymatic activity of CD73 by measuring adenosine production following incubation with ATP.

#### Distribution of HLA-G isoforms in semen fractions

Screening pooled SEV and VDSP fractions for classical and nonclassical HLA molecules (HLA-A, HLA-B, HLA-C, HLA-DR, HLA-DP, HLA-DQ, HLA-E, and HLA-G) revealed detectable HLA-G, which occurs in different isoforms of varying molecular weight (Clements *et al.*, 2005; Carosella *et al.*, 2008). This finding aligns with previous reports describing soluble HLA-G isoforms in semen (Schallmoser *et al.*, 2025). Notably, two distinct bands were observed in SEV but not in VDSP (Supplementary Fig. S1), suggesting that SEV also contains a higher molecular weight membrane-bound isoform as well as smaller, soluble isoforms. None of the other screened HLA molecules were present in SEV or VDSP.

**Figure 1.**
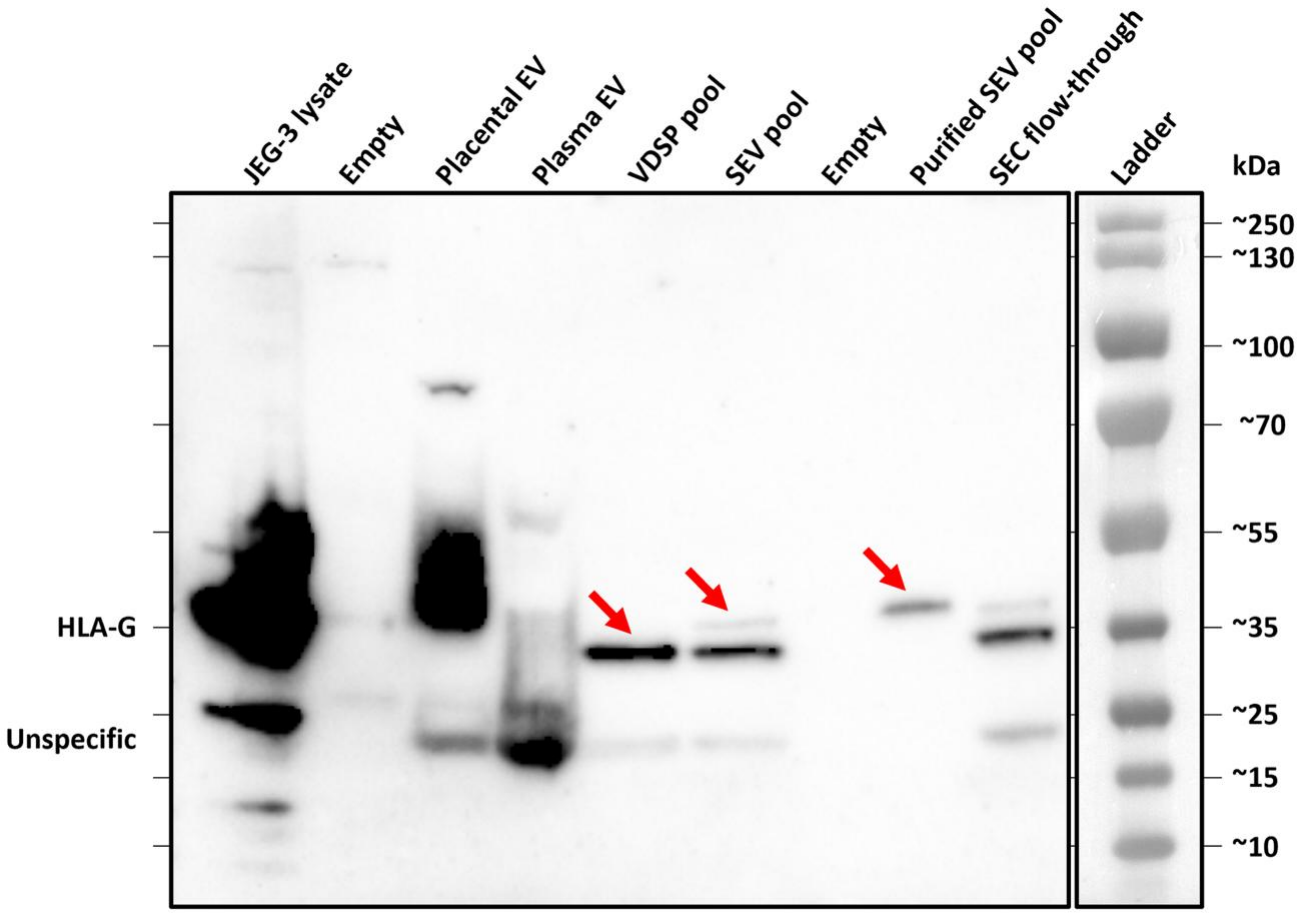
**Semen extracellular vesicles (SEVs) and vesicle-depleted semen plasma (VDSP) contain human leukocyte antigen G (HLA-G)**. Different isoforms of HLA-G are present in semen. Approximately 30 µg of total protein from each sample were loaded for SDS–PAGE followed by western blotting. Samples included lysates from JEG-3 cell line (HLA-G positive control), extracellular vesicles (EVs) from a placenta (HLA-G positive control), EVs from plasma (HLA-G negative control), pools of VDSP and SEV, highly purified SEV samples, and SEC flow-through (eluent) collected after SEC of the SEV sample, representing smaller molecules co-present in the unpurified SEV pool. Red arrows indicate the two HLA-G isoforms present in semen. SEC, size-exclusion chromatography.

To confirm this differential distribution, SEV underwent additional purification by SEC (70 nm columns) to further remove small proteins not associated with SEV. Following collection of the SEV fractions, the later SEC flow-through (eluent), enriched for smaller non-EV molecules was collected. The purified SEV fraction was reconcentrated prior to analysis. Western blotting of the purified SEV sample revealed a clear distinction between HLA-G isoforms associated with SEV and those present in the VDSP fraction (Fig. 1).

This experiment was repeated across multiple individuals to assess inter-individual variability in HLA-G content. The higher-molecular-weight isoform, likely corresponding to the membrane-bound form, varies in concentration across SEV samples purified from one total semen donation (Supplementary Fig. S2). These observations raise the possibility that variability in HLA-G could influence immune modulation in the semen recipient. Future studies will be needed to determine whether this variation contributes to differences in immune compatibility and ultimately reproductive success between partners.

#### Localization and activity of semen ectonucleotidases

We next examined the presence of the ectonucleotidases CD39 and CD73 in SEV and VDSP. These molecules have been implicated in immunosuppression within the tumor microenvironment (Liu *et al.*, 2025) and have been reported in tumor-derived EVs (Wang *et al.*, 2021; Whiteside *et al.*, 2025). They sequentially hydrolyze extracellular ATP to adenosine, a metabolite with well-established immunoregulatory properties. Our western-blot analysis revealed that CD73 (reported molecular weight at 63 kDa in tumor-derived small EVs; Lu *et al.*, 2022) was present in the SEV fraction and was absent from the VDSP fraction (Supplementary Fig. S3). There was no detectable CD39 band in either semen fraction.

**Figure 2.**
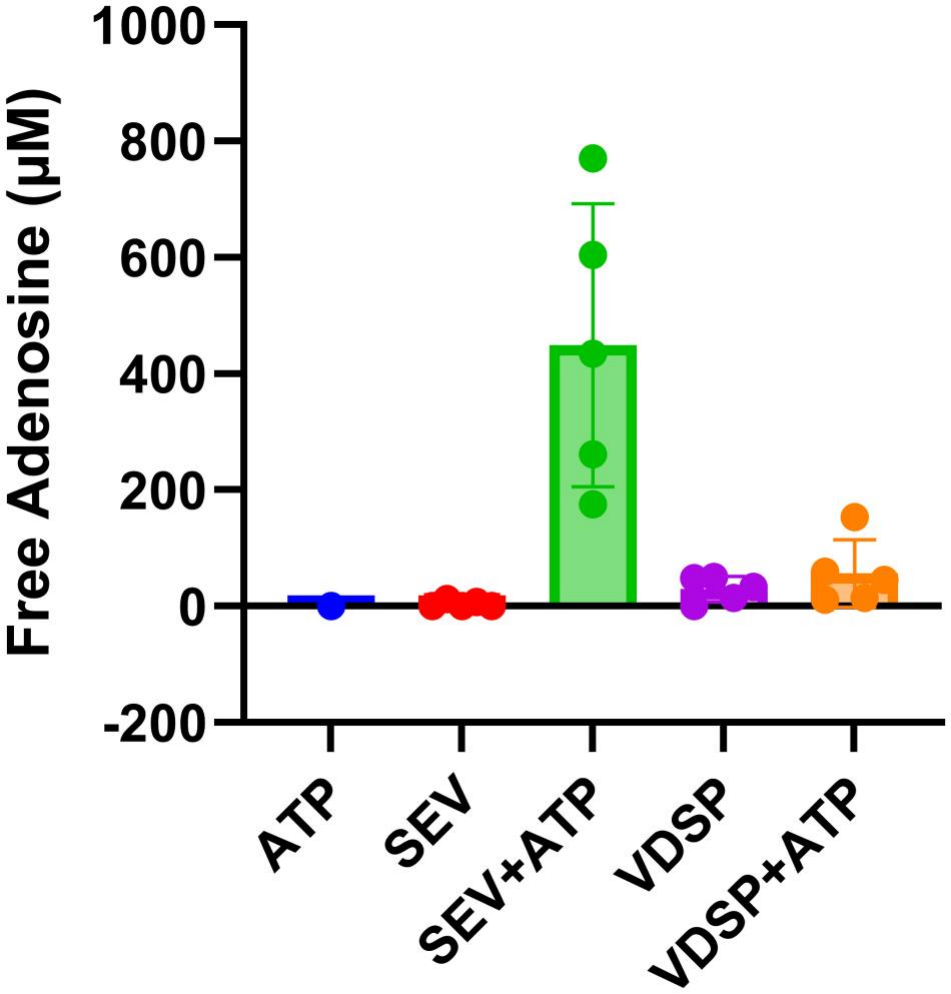
**Semen extracellular vesicles (SEVs) but not vesicle-depleted semen plasma (VDSP) samples generate immunosuppressive adenosine from adenosine triphosphate (ATP)**. Results from five individuals are shown. Bars and lines indicate means and SDs.

To assess the functional activity of CD73, which is responsible for the production of free adenosine from AMP, ATP was incubated with SEV and VDSP fractions, and adenosine production was measured. Because CD73 hydrolyzes AMP rather than ATP, ATP consumption in our assay is unlikely to be mediated by CD73 alone. In the absence of detectable CD39, this activity could instead reflect co-purified upstream ectonucleotidases/phosphatases capable of converting ATP to AMP, such as members of the ectonucleotide pyrophosphatase/phosphodiesterase (ENPP) family (Winzer *et al.*, 2024) (e.g. ENPP3 has been reported in semen-derived Evs; Zhang *et al.*, 2020). However, ENPP expression or activity was not directly assessed in our semen EV preparations. Except for one VDSP sample, none increased adenosine concentration; in contrast, all SEV samples increased adenosine concentration (Fig. 2), indicating functional CD73 activity that produces the immunologically active end metabolite. These findings suggest that SEV may modulate recipient cell function by raising extracellular adenosine levels.

### Inter-individual variation in semen-induced tolerance response

#### Rationale and experimental design

Building on our earlier findings (Paktinat *et al.*, 2025), we selected key tolerance-associated immune signatures identified in *in vitro*-differentiated Tregs induced by each semen fraction to compare tolerance responses across multiple semen donor-recipient pairs. This setup models the immunological events following sexual intercourse. After 7 days of coculture, we assessed the emergence of Forkhead box P3 (FOXP3)+ CD25Hi CD127Lo CD4+ T cells, referred to as Tregs hereafter. We further assessed Treg phenotypic profiles, including proliferating Ki-67+ Tregs and subsets of Tregs expressing suppressive markers, including the co-expression of programmed cell death protein 1 (PD-1) and cytotoxic T-lymphocyte-associated protein 4 (CTLA-4), or T-cell immunoreceptor with Ig and ITIM domains (TIGIT)+, as well as amphiregulin (AREG)+ Tregs. The latter are associated with tissue repair functions and may be involved in decidual preparation for implantation and placentation (Lysiak *et al.*, 1995; Fang *et al.*, 2021; Lv *et al.*, 2022; Raheem *et al.*, 2025). We also assessed the generation of LAG-3+ CD49b+ CD25Lo FOXP3− CD4+ T cells, referred to as type 1 regulatory T (Tr1)-like cells, which are considered to be alloantigen-specific regulatory T cells (Chen *et al.*, 2021). The gating strategy used to identify each of these CD4+ T cell populations is shown in Supplementary Fig. S4.

#### Tolerogenic effect size of each semen fraction in each recipient

**Figure 3.**
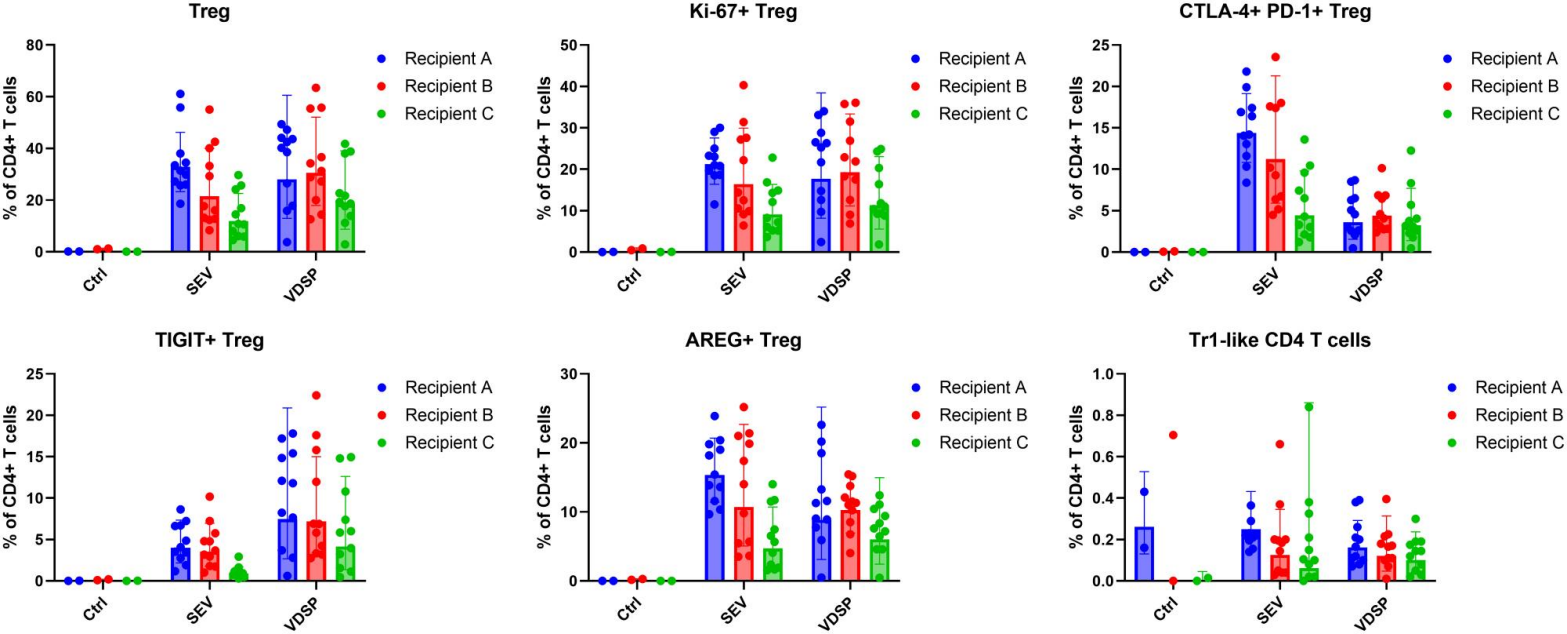
**Tolerogenic effect size of each semen fraction in each recipient**. Regulatory T cell (Treg) populations are shown as a percentage of total CD4+ T cells following stimulation with each semen fraction in three recipients (A–C). Bars represent the geometric mean±geometric SD across 11 independent semen donors, and dots are averaged data from replicate wells. Phosphate-buffered saline (PBS)-treated cultures served as background controls. Treg: FOXP3+ CD25Hi CD127Lo CD4+ T cells, Tr1-like: LAG-3+ CD49b+ CD25Lo FOXP3− CD4+ T cells. SEVs, semen extracellular vesicles; VDSP, vesicle-depleted semen plasma; CTLA-4, cytotoxic T-lymphocyte-associated protein 4; PD-1, programmed cell death protein 1; TIGIT, T-cell immunoreceptor with Ig and ITIM domains; AREG, amphiregulin; Tr1, type 1 regulatory T cell; FOXP3, Forkhead box P3.

We quantified the induction of each Treg population in response to each semen fraction. Figure 3 summarizes these results as the frequency of each population within total CD4+ T cells. Naïve CD4+ T cells cultured alone, or with SEV or VDSP in the absence of MoDCs, failed to survive to the analysis timepoint (data not shown), consistent with a requirement for DC-derived cues for T-cell activation and survival in this system. In the PBS control condition, Tregs were essentially undetectable across most populations; the sole exception was the Tr1-like subset, for which recipient A exhibited a measurable baseline frequency even in the absence of semen-derived stimulation. Across all recipients and fractions, the observed frequencies spanned a wide dynamic range: total Tregs, 2.88% to 63.4%; Ki-67+ Tregs, 1.87% to 40.3%; PD-1+ CTLA-4+ Tregs, 0.46% to 28.6%; TIGIT+ Tregs, 0.31% to 22.4%; AREG+ Tregs, 0.475% to 25.2%; and Tr1-like cells, 0.0000502% to 1.06%. Stratifying by recipient revealed marked inter-individual variability: recipient C (green dots) consistently displayed the lowest frequencies across all populations (a low-responder phenotype), whereas recipients A (blue dots) and B (red dots) showed broadly comparable responses and were more similar to each other than to recipient C.

#### Normalization strategy to visualize donor-recipient pair variations

To compare trends across donor–recipient pairs, we first adjusted for each recipient’s baseline by subtracting the frequency observed in the PBS-treated control. We then scaled the adjusted values to the largest increase observed across all three recipients for each cell population. Supplementary Fig. S5 presents a summary of the normalized dataset depicted in the heatmaps for each cell population.

#### Recipient-influenced patterns

**Figure 4.**
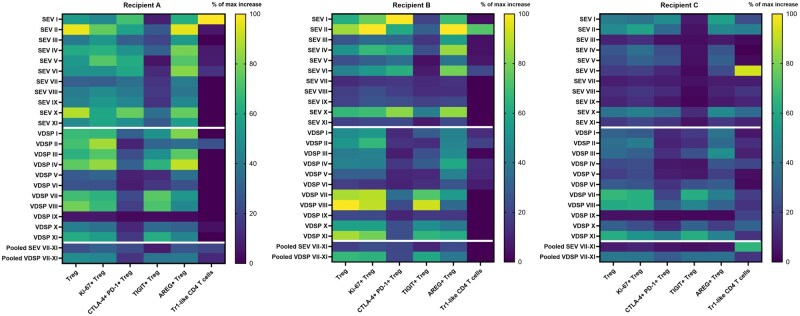
**Heatmaps of increases in tolerance-associated regulatory T cell (Treg) signatures induced by semen extracellular vesicles (SEVs) and vesicle-depleted semen plasma (VDSP) across 11 semen donors**. Population frequencies were normalized to the recipient-specific phosphate-buffered saline (PBS) control and then max-scaled for each signature using the highest increase observed across all recipients for a given semen fraction (either SEV or VDSP). Each heatmap displays increase in the frequencies of six T cell phenotypes (*x*-axis) for the indicated recipient. For SEV and VDSP samples, numbering denotes corresponding pairs. ‘Pooled’ SEV and VDSP were generated by combining samples from five donors. CTLA-4, cytotoxic T-lymphocyte-associated protein 4; PD-1, programmed cell death protein 1; TIGIT, T-cell immunoreceptor with Ig and ITIM domains; AREG, amphiregulin; Tr1:, type 1 regulatory T cell.

Recipient C showed the lowest Treg induction (Fig. 4) and minimal Ki-67+ Tregs. In contrast, recipient B consistently exhibited the strongest Treg and Ki-67+ Treg induction, as well as the highest frequencies of TIGIT+, PD-1+ CTLA-4+, and AREG+ Tregs. Recipient A showed moderate Treg induction across multiple semen donors. These data show that a particular recipient can be hyporesponsive to a wide array of semen donors across both semen fractions tested, for example recipient C. Other recipients are more selectively responsive to certain semen donors, or more responsive to the majority of semen donors and semen fractions.

#### Donor fraction (SEV or VDSP)-influenced patterns

Certain semen samples induced comparable responses across recipients; for example, SEV II elicited consistently high responses across the responding recipients (e.g. A and B, in Figs 3 and 4) and cell populations. In contrast, some samples revealed donor-recipient specificity; for example, VDSP VII and VDSP VIII strongly induced Treg in recipient B but had a lower impact on recipients A and C. Conversely, SEV VII and SEV VIII elicited stronger responses in recipient A than in recipients B and C. In addition, SEV VII was among the least tolerogenic semen fractions across recipients, while VDSP from the same donor was among the more tolerogenic. Most VDSP samples, except VDSP IX, induced proliferating (Ki-67+) Tregs and TIGIT+ subsets, while SEV samples preferentially promoted PD-1+ CTLA-4+ and AREG+ Tregs, particularly in recipients A and B. Pooling, as might be expected, dampened the highest responses and moved most cellular signatures toward the median response. Together, these data show that a particular semen fraction from a particular donor may consistently fail to induce a tolerogenic response across several recipients. However, when considering both fractions, semen from each donor appeared to have at least some tolerogenic effect.

#### Combined fractions analysis

**Figure 5.**
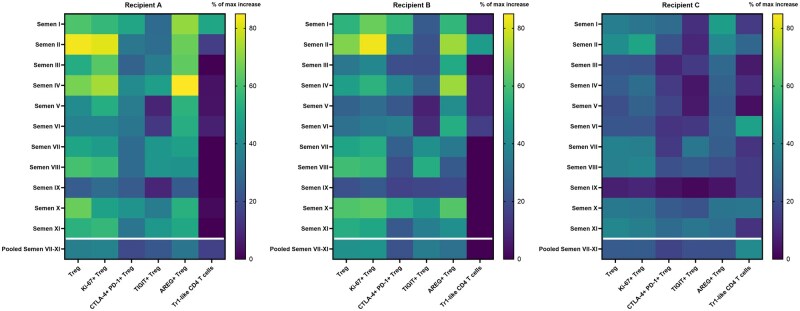
**Heatmaps of increases in tolerance-associated regulatory T cell (Treg) signatures in response to whole semen modeled by combined semen extracellular vesicles (SEVs) and vesicle-depleted semen plasma (VDSP) fractions**. Because SEV and VDSP showed differential tolerogenic effects, we combined donor-matched SEV and VDSP responses to estimate their overall impact and better approximate physiological exposure to whole semen. Each heatmap displays six tolerance-associated Treg signatures (*x*-axis) for the indicated recipient. CTLA-4, cytotoxic T-lymphocyte-associated protein 4; PD-1, programmed cell death protein 1; TIGIT, T-cell immunoreceptor with Ig and ITIM domains; AREG, amphiregulin; Tr1, type 1 regulatory T cell.

Because we found that the two semen fractions had differential effects on tolerance induction and that at least one fraction from each donor appeared tolerogenic, we wanted to assess the combined impact of both fractions. This also simulates physiological conditions better, because during sexual intercourse, a recipient gets exposed to the whole semen and not fractions of it. We averaged data from SEV and VDSP fractions from individual donors and reassessed immune responses as if the fractions had not been separated. Figure 5 summarizes these results. Recipient A again showed the highest induction, with recipient B intermediate and recipient C the lowest. In terms of semen donor potency across recipients, donors II, IV, and X emerged as strong inducers of Treg differentiation in recipients A and B. Donor IX consistently showed low induction across all recipients. We observed some evidence of Tr1-like cells, for example SEV I with recipient A, SEV II with recipient B, and SEV VI with recipient C (Fig. 4).

Altogether, donor-recipient specific-pair effects were less evident overall, though one example may be semen donor III, who showed greater potency in promoting Treg differentiation in recipient A than in recipient B. This analysis confirmed that when both fractions were virtually combined, semen from almost all donors exhibited tolerogenic effects with remarkable interindividual variation.

### Potential antigen-specific memory-like activation in semen-induced Treg

#### Rationale and experimental design

To explore whether semen induced Tregs exhibit features of antigen-specific memory-like activation, we implemented a re-exposure protocol combined with an AIM assay. The AIM assay is a flow cytometry-based method used to detect surface marker expression associated with TCR-dependent activation (Tippalagama *et al.*, 2023). Although it does not directly confirm antigen specificity, the co-expression of specific surface markers has been used to identify cells likely activated by their cognate antigens (Lemieux *et al.*, 2024; Zheng *et al.*, 2025). A recent refinement of the assay introduced the combination of CD137 upregulation and CXCR4 downregulation as a reliable surrogate for antigen-specific activation (Zheng *et al.*, 2025).

**Figure 6.**
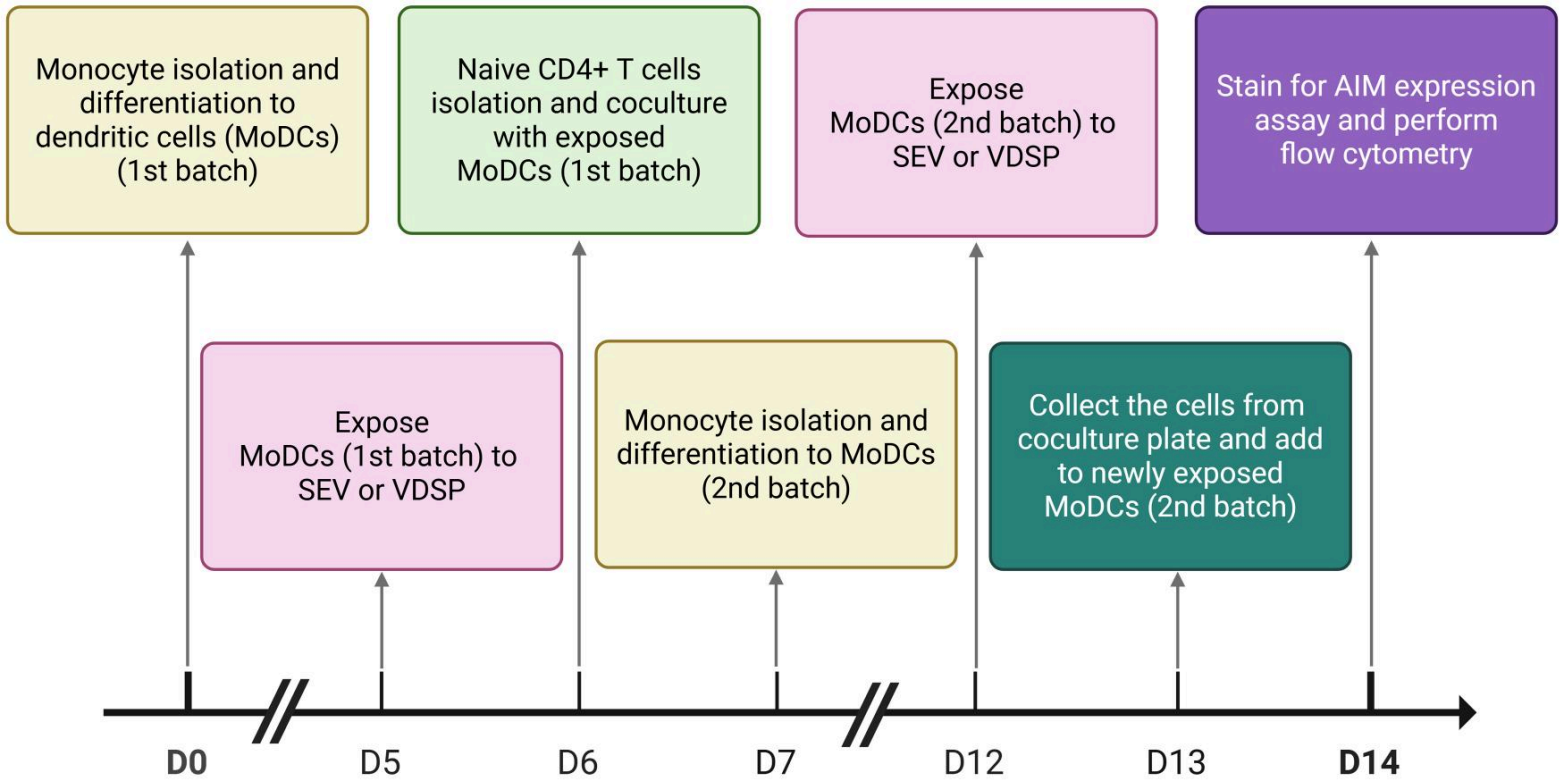
**Overview of the activation-induced marker (AIM) expression assay workflow**. MoDCs: monocyte-derived dendritic cells. Created in BioRender. Paktinat, S. (2026) https://BioRender.com/z5cmghe.

For the re-exposure, we collected cells on Day 7 from the cultures described above. In parallel, fresh autologous MoDCs were loaded with SEV or VDSP fractions and then incubated overnight (Days 7–8) with the harvested cells. Re-exposure was considered ‘linked’ when cells were re-stimulated with the same semen sample used for priming. We also included an ‘unlinked’ condition in which cells were re-stimulated with fractions from a different donor than the one used for priming. SEV and VDSP fractions from four semen donors were tested on the same recipient, and AIM induction was assessed specifically within the Treg (FOXP3+ CD25Hi CD127Lo CD4+ T cells) population. Figure 6 summarizes the AIM assay workflow and experimental timeline.

#### Immediate effect of SEV and VDSP re-exposure on canonical Treg markers

**Figure 7.**
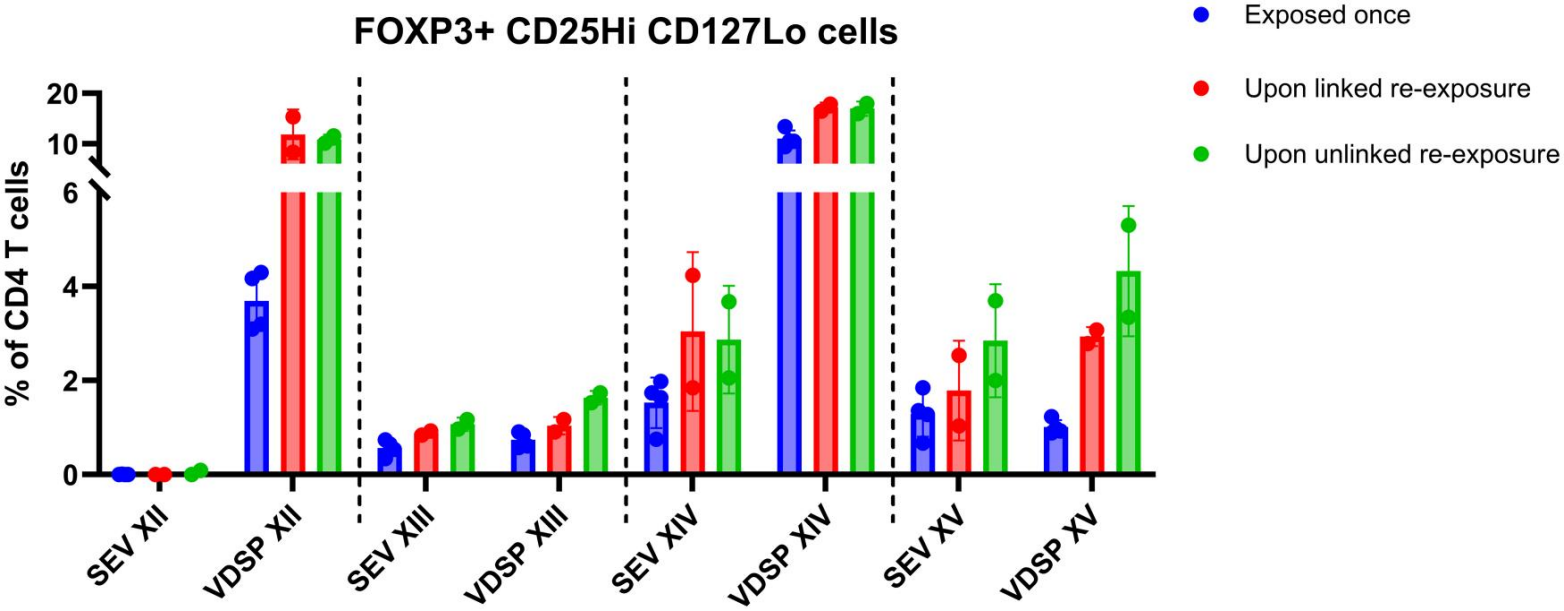
**Frequencies of FOXP3+ regulatory T cells (Tregs) increase upon second exposure to individual semen fractions**. Results from unique recipient cell cultures (recipient D) are shown as individual dots, bars and lines indicate mean and SD. Exposed once: cells harvested at Day 7 at the end of the initial monocyte-derived dendritic cells (MoDCs)/naïve CD4+ T cell co-culture, with no further stimulation during Days 7–8. Linked re-exposure: Day 7 harvested cells re-stimulated for 24 h (Days 7–8) with semen extracellular vesicles (SEVs) or vesicle-depleted semen plasma (VDSP) fractions from the same semen sample used for priming. Unlinked re-exposure: Day 7 harvested cells re-stimulated for 24 h (Days 7–8) with SEV/VDSP fractions from a different semen sample than that used for priming. FOXP3, Forkhead box P3.

To evaluate the stability of Treg lineage markers, we first evaluated whether overnight re-exposure to MoDCs loaded with different semen components altered the frequency of FOXP3+ CD25Hi CD127Lo CD4+ Treg cells, as a readout of short-term (overnight) induction of the canonical Treg markers FOXP3 and CD25. VDSP XII, VDSP XV, and to a lesser extent VDSP XIV, induced increased frequencies of FOXP3+ CD25Hi CD127Lo CD4+ T cells upon re-exposure (Fig. 7). SEV fractions had minimal impact. In VDSP XII- and VDSP XIV-primed cells, the magnitude of increase was similar regardless of whether the re-exposure was linked or unlinked. In VDSP XV, the unlinked re-exposure had a stronger effect than the linked exposure. This suggests that the response may reflect recognition of antigens broadly shared across semen samples, or alternatively a nonspecific activation of T cells.

To assess dose sensitivity, we repeated the experiment using reduced concentrations of VDSP XII and VDSP XIV. The pattern remained consistent: VDSP XII continued to induce an increase in CD25 and FOXP3 expressions, although the overall magnitude was reduced with lower concentrations (Supplementary Fig. S6). In contrast, VDSP XIV did not exhibit a strong concentration-dependent effect and maintained relatively high CD25 and FOXP3 expression even at reduced concentrations.

These findings suggest that certain semen samples more potently stimulate upregulation of lineage commitment markers related to regulatory T cells, potentially boosting the resulting population of Tregs. The data also indicates that unlinked re-exposures were as effective as linked re-exposures.

#### Antigen-specific activation potential assessed by AIM assay

**Figure 8.**
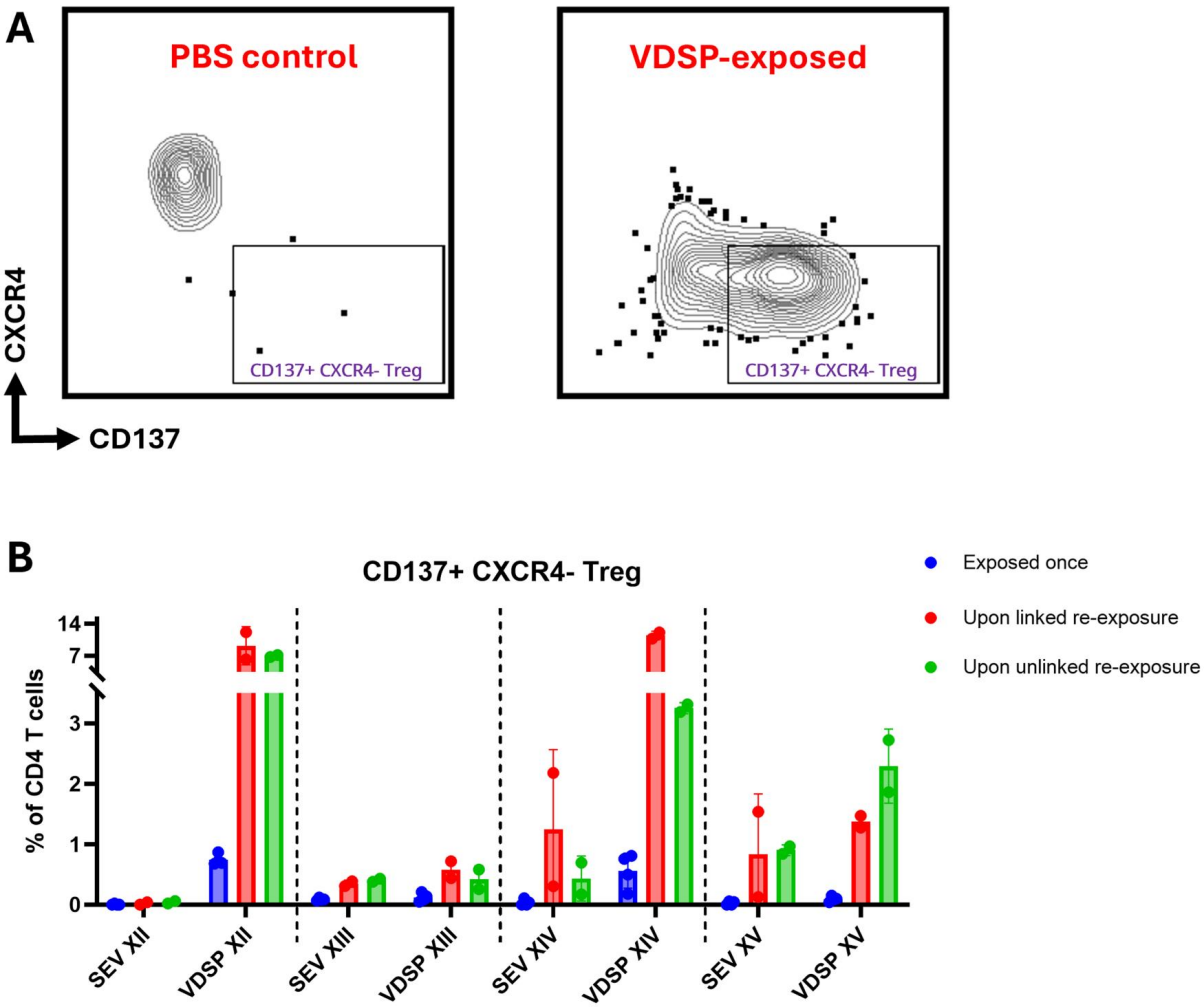
**Activation-induced marker (AIM) expression in regulatory T cells (Tregs) is enhanced in response to re-exposure to the same semen extracellular vesicles (SEVs) or vesicle-depleted semen plasma (VDSP) compared to re-exposure with unlinked semen fractions**. (**A**) CD137+ CXCR4− gating on FOXP3+ CD25Hi CD127Lo CD4+ T cells (Treg). (**B**) Frequencies of CD137+ CXCR4− Tregs are shown. Results from unique recipient cell cultures (recipient D) are shown as individual dots, bars, and lines indicate mean and SD. Exposed once: cells harvested at Day 7 at the end of the initial monocyte-derived dendritic cells (MoDCs)/naïve CD4+ T-cell co-culture, with no further stimulation during Days 7–8. Linked re-exposure: Day 7 harvested cells re-stimulated for 24 h (Days 7–8) with SEV/VDSP fractions from the same semen sample used for priming. Unlinked re-exposure: Day 7 harvested cells re-stimulated for 24 h (Days 7–8) with SEV/VDSP fractions from a different semen sample than that used for priming. PBS, phosphate-buffered saline; FOXP3, Forkhead box P3.

To evaluate potential alloantigen-specific Treg activation, we assessed AIM expression using CD137+ CXCR4− gating (gate indicated in Fig. 8A). As shown in Fig. 8B, VDSP XII, VDSP XIV, and VDSP XV consistently increased the frequency of CD137+ CXCR4− Treg following both linked or unlinked re-exposure. Among these, VDSP XIV in the linked condition induced the highest AIM+ Treg frequency, whereas the unlinked re-exposure with VDSP XIV resulted in a noticeably lower response. VDSP XII showed similar AIM+ Treg frequencies in both linked and unlinked conditions, comparable in magnitude to the VDSP XIV responses. The opposite was seen for VDSP XV, where the unlinked response was higher than the linked one. SEV XIV and SEV XV also induced CD137+ CXCR4− Treg, though the data points were less consistent across replicates.

To confirm these observations, we repeated the experiment using VDSP XII and VDSP XIV. In both cases, re-exposure to the same donor’s sample led to increased frequencies of CD137+ CXCR4− Tregs, consistent with the initial experiment (Supplementary Fig. S7). For VDSP XII, reducing the concentration resulted in diminished CD137+ CXCR4− Treg frequencies, and responses were similar between linked or unlinked re-exposures. In contrast, reducing the VDSP XIV concentration by half did not alter the frequency of CD137+ CXCR4− Tregs in the linked condition. Although VDSP XIV-primed cells also showed elevated AIM+ Treg frequencies upon unlinked re-exposure, the response was less pronounced compared to the linked condition. This pilot *in vitro* study suggests that certain semen samples may promote memory-like activation in Treg, with some evidence pointing toward donor-specific effects that could reflect underlying antigenic influences.

## Discussion

Here, we tested the hypothesis that inter-individual variation exists in the immunoregulatory composition of semen, which in combination with intrinsic recipient factors, shape tolerogenic immune responses. We found that while there was some variation in induction of tolerance in individual semen fractions, overall recipient-level susceptibility to semen-mediated tolerance seemed to play a larger role in regulatory T cell generation.

Previous human studies have examined semen-induced immune responses in the cervix (Sharkey *et al.*, 2012) and in the endometrium (Catalini *et al.*, 2024), where ascending semen signals may contribute to tissue preparation for implantation. Although semen is deposited in the lower genital tract in humans, its signals may reach the uterus through several routes, including uterine peristalsis (Kunz *et al.*, 1996), sperm-associated transport of factors such as TGF-β (Chu *et al.*, 1996), and counter-current transfer from the vaginal venous circulation to the uterine artery (Cicinelli and de Ziegler, 1999; Einer-Jensen and Hunter, 2005). In support of uterine responsiveness to semen signals, human endometrial epithelial and stromal cells exposed to semen factors *in vitro* have been shown to induce genes associated with leukocyte recruitment, endothelial cell migration, tissue remodeling, and cell survival (Gutsche *et al.*, 2003; Ochsenkuhn *et al.*, 2008; Chen *et al.*, 2014). In a recent study, van den Berg *et al.* (2025) examined the direct effects of semen plasma on endometrial epithelial organoids from fertile and sub-fertile individuals. Despite interindividual heterogeneity, transcriptomic analysis identified a donor-independent epithelial response to semen plasma, with differentially expressed genes spanning multiple biological processes, including pathways related to immune regulation and endometrial receptivity. The effects of isolated semen EVs on the endometrium have also been explored *in vitro*. For example, semen EVs can bind to human endometrial stromal cells and enhance decidualization (Rodriguez-Caro *et al.*, 2019), and have been reported to increase endometrial receptivity via the LIF pathway (Wang *et al.*, 2024). Together, these studies suggest that semen exposure elicits a coordinated immune response in the recipient’s reproductive tract, with evidence of effects extending to the endometrium.

Apart from direct effects on the endometrium, semen has a well-established effect on the vagina and cervix, with substantial influx of leukocytes and local upregulation of cytokines and chemokines (Sharkey *et al.*, 2007, 2012; Jewanraj *et al.*, 2020). Evidence from human and animal studies suggests that these signals initiate immune and tissue-remodeling programs with potential effects on fertility, pregnancy outcomes, and offspring health (Robertson and Sharkey, 2016). Semen contains EVs (SEV) and soluble factors (VDSP) enriched in immunoregulatory molecules such as TGF-β, HLA-G, prostaglandins, and the ectonucleotidase CD73. A few studies have revealed heterogeneity in concentration and types of immunomodulatory factors in semen and their correlations with clinical outcomes. Overall, TGF-β levels were reduced in semen resulting in live births following ART (Schallmoser *et al.*, 2025), while another study assessing TGF-β isoforms found TGF-β2 concentrations were significantly higher, and TGF-β3 concentrations significantly lower, in individuals with reduced semen quality compared to fertile sperm donors (Nilsson *et al.*, 2020). Another report also links a specific TGF-β3 gene variant to infertility (Drozdzik *et al.*, 2015). For HLA-G, though variation in semen has been reported (Dahl *et al.*, 2014; Schallmoser *et al.*, 2019, 2025; Nilsson *et al.*, 2020), the clinical relevance remains unclear, particularly with respect to fertility outcomes. A few studies found no association between semen soluble HLA-G levels and pregnancy outcomes (Schallmoser *et al.*, 2019; Nilsson *et al.*, 2020). However, in couples undergoing ART, absolute concentrations of semen HLA-G and TGF-β were significantly lower in semen from the partners resulting in live birth than those associated with unsuccessful outcomes (Schallmoser *et al.*, 2025). This suggests that semen immune regulation may require a finely balanced signal: sufficient to support tolerance and endometrial receptivity, but not excessive or poorly coordinated in a way that compromises endometrial selectivity.

EVs are under active investigation as regulators of reproductive biology and immunology (Vojtech *et al.*, 2014, 2019; Marques de Menezes *et al.*, 2020; Wang *et al.*, 2020; Tamessar *et al.*, 2024, 2026; Zhang *et al.*, 2024; Okeoma *et al.*, 2025; Paktinat *et al.*, 2025). One function of SEV, both before and after ejaculation, is to deliver cargo to spermatozoa, enabling sperm maturation, protection from oxidation, and the acrosome reaction (Burden *et al.*, 2006; Ronquist, 2015). They also have potent effects on recipient immune cells and modulate inflammation in the genital tract (Marques de Menezes *et al.*, 2020; Paktinat *et al.*, 2021). As in other studies, we observed that SEV contain different isoforms and variable levels of HLA-G, a well-established ligand for inhibitory receptors which induces tolerogenic DCs (Ristich *et al.*, 2005; Mao *et al.*, 2023). We also found that SEV carry functional ectonucleotidase CD73 that dephosphorylates ATP to create free adenosine. The generation of extracellular adenosine via EV-associated CD73, and the downstream signaling it drives, is well established in human immunologic contexts (Clayton *et al.*, 2011; Schneider *et al.*, 2021). Building on these observations, we propose that semen EVs exert some of their immunomodulatory effects through an extracellular adenosinergic mechanism at the deposition site. This mechanism is biologically plausible because extracellular ATP is typically maintained at very low levels but can rise sharply during tissue stress, inflammation, or mechanical irritation, thereby providing substrates for adenosine generation (Savio *et al.*, 2018; Giuliani *et al.*, 2020). Notably, vaginal epithelium, like other barrier tissues, exhibits mechanosensitive ATP release, and intercourse can be associated with epithelial microtrauma (Button and Boucher, 2008; Harroche *et al.*, 2020; Borgogna *et al.*, 2023), suggesting a route by which coitus could transiently elevate luminal extracellular ATP and potentiate SEV-linked adenosinergic effects. This hypothesis can be tested in human mucosal explants and organoid-based platforms with quantitative measurements of ATP and adenosine flux.

In addition, SEV directly impairs antigen presentation by APCs, weakening downstream antiviral memory T cell responses (Vojtech *et al.*, 2019). It has been reported that SEVs can act directly on T cells and promote differentiation toward a regulatory phenotype (Zhang *et al.*, 2024). We previously examined the effects of SEVs and VDSP on recipient DCs and their subsequent capacity to shape naïve CD4+ T cell differentiation, finding that semen induces a tolerogenic phenotype in APCs which drives differentiation of Tregs from naïve T cells (Paktinat *et al.*, 2025). Though the antigen-specificity of these Tregs was not formally established, epidemiological studies show that cumulative exposures to a specific partner’s semen are protective against PE (Saftlas *et al.*, 2014; Galaviz-Hernandez *et al.*, 2018). This suggests that seeding a pool of paternal antigen-specific regulatory T cells may help to support healthy pregnancies. Here, using a pairing matrix of SEV and VDSP fractions from 11 semen donors with immune cells from three randomly selected recipients, we observed marked donor- and recipient-dependent variability in the induction of tolerance-associated regulatory T cell phenotypes. Though the number of recipient samples was limited, our data suggest this variability was more strongly shaped by recipient factors than by semen donor factors: recipients differed in their capacity to acquire tolerogenic signatures, whereas donor-dependent effects were comparatively less pronounced. This may be because the immune setpoint in any individual is influenced by a multitude of factors including genetic differences, early life events, stress, age, diet, environmental exposures, etc., while the generation of semen is less susceptible to influence (Liston *et al.*, 2021; Balcerowska and Kwasnik, 2025). Notably, we also observed donor-specific compartmentalization, in which one fraction from a given donor was consistently non-reactive (for example, VDSP VI in Fig. 4), while the paired fraction from the same donor remained reactive (SEV VI in recipients A and B).

We observed enhanced canonical Treg marker expression upon re-encounter with previously experienced semen under both linked and unlinked exposure conditions. We speculate that this may reflect an antigen-driven adaptive response that builds with repeated semen exposure. In our experiments, given the short timeframe, this may not yet represent true immunological memory, rather, it could reflect a lower activation threshold on re-exposure, or possibly further expansion of initially primed Tregs upon restimulation. Whether semen exposure elicits truly antigen-specific and memory-like regulatory T cell responses, or whether the observed changes reflect non-specific bystander activation remain as key conceptual questions. The gold standard for demonstrating antigen specificity and clonal memory would require isolation of responding T cells, high-resolution TCR sequencing, and longitudinal tracking of clonotypes after repeated exposure to defined semen antigens, approaches that are technically demanding and currently feasible only in limited human settings. In this study, we instead used surface activation markers that are rapidly changed following TCR engagement (AIM assay) as a pragmatic proxy. We found evidence for an antigen-specific component in response to VDSP that was less apparent in the SEV fraction. This pattern suggests that soluble or low-molecular weight components in semen plasma (such as peptides, free proteins, or small complexes) may more readily access APCs and drive classical TCR-mediated activation, whereas SEVs may act via a combination of cargo delivery and pattern-recognition pathways. Nevertheless, without TCR sequencing or peptide-MHC multimer staining, our data should be interpreted as evidence for boosted responses on secondary exposure rather than definitive proof of antigen-specific memory.

One limitation of our study is that we used peripheral blood from a small number of recipients who were not selected based on reproductive age, documented fertility status, or other clinical criteria. Circulating cells are not exposed to the tissue environment or local hormonal context, which could influence the response to semen. As a result, we cannot link the observed responses to implantation success or pregnancy outcomes. However, from an immunologic standpoint, the capacity to mount TCR-dependent responses and to expand regulatory subsets should be an intrinsic characteristic, and peripheral blood remains a practical and widely used compartment for studying antigen-specific T cell responses in humans. Thus, while the age and reproductive history of our donors may influence the magnitude of responses, the fundamental qualitative features of TCR engagement and Treg induction observed here are still informative. Another limitation is that due to limited access to semen samples from the same individuals, we could not link levels or activity of specific cargo molecules like CD73 or HLA-G to semen tolerance-inducing function, which limits interpretation of this finding as a confirmed mechanism. We also used averaged results from SEV and VDSP to assess the overall effect of complete semen, rather than directly testing unfractionated semen. Whole semen may exhibit overall different effects than each fraction separately.

**Figure 9.**
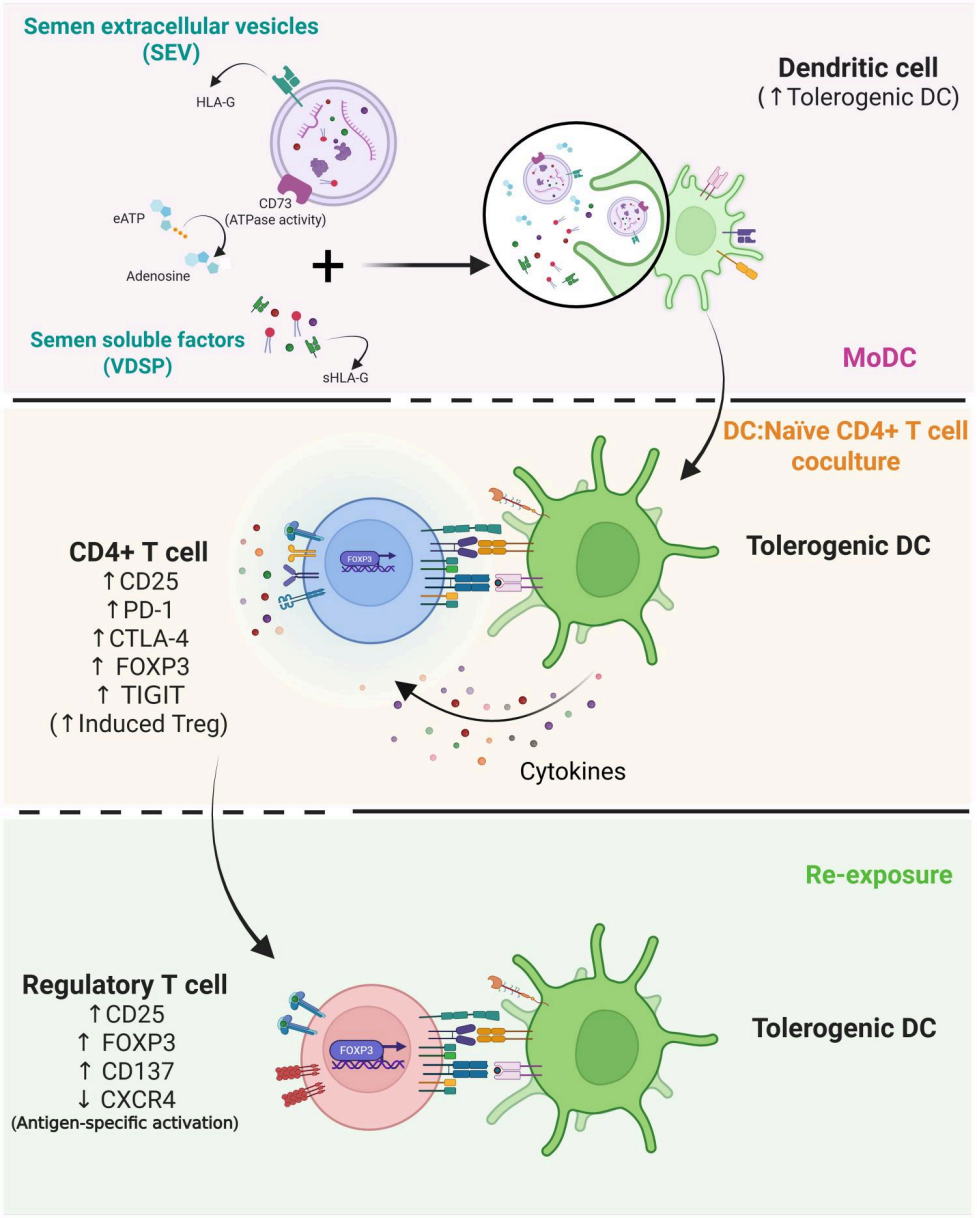
**Proposed model of semen-driven pathway leading to tolerance induction in the recipient**. Semen contains extracellular vesicles (SEVs) and soluble factors (VDSP) enriched in human leukocyte antigen G (HLA-G) and the ectonucleotidase CD73, which catalyzes extracellular adenosine generation (from eATP) and promotes a tolerogenic program in recipient dendritic cells (DCs). These tolerogenic DCs prime naïve CD4+ T cells to differentiate into regulatory T cells (Tregs) expressing tolerance-associated signature markers. Upon subsequent exposure to semen, newly induced tolerogenic DCs presenting the same antigens reactivate antigen-specific Tregs, which modulate immune responses and help establish a permissive environment for successful embryo implantation. eATP, extracellular adenosine triphosphate; sHLA-G, soluble human leukocyte antigen G. MoDCs, monocyte-derived dendritic cells; CTLA-4, cytotoxic T-lymphocyte-associated protein 4; PD-1, programmed cell death protein 1; TIGIT, T-cell immunoreceptor with Ig and ITIM domains; AREG, amphiregulin; Tr1, type 1 regulatory T cell; FOXP3, Forkhead box P3. Created in BioRender. Paktinat, S. (2026) https://BioRender.com/z5l3dtd.

Despite these limitations, we propose that our data provide a conceptual framework for semen-immune interactions in human reproduction. Our proposed model for semen-mediated immune tolerance is summarized in Fig. 9. Rather than viewing ‘immunological compatibility’ as a binary property of a couple, we see it as a multidimensional landscape defined by (i) the composition and immunoregulatory capacity of semen, including SEV cargo; (ii) the baseline and inducible regulatory potential of the immune system including the capacity to generate and maintain antigen-specific Treg populations; and (iii) the history of previous sexual exposures. This framework underscores the importance of integrating multiple immunological factors when considering the etiology of complex reproductive disorders. Accordingly, some cases of otherwise unexplained infertility, recurrent implantation failure, or recurrent pregnancy loss may reflect positioning within this landscape: for example, semen with limited Treg-inducing capacity in a recipient prone toward inflammatory responses, or conversely, a highly tolerogenic pairing that compromises adequate anti-infection defense. Looking forward, high-dimensional multiomics approaches combined with TCR repertoire analysis may help identify ‘signatures’ of beneficial versus detrimental semen-induced immune responses.

Future work should prioritize translating these findings into well-defined clinical and experimental settings. First, samples from couples with long-standing sexual relationships could be screened using the design used here to compare responses within couples to those from unrelated pairs. Second, well-characterized cohorts of fertile couples, alongside cohorts affected by adverse pregnancy outcomes such as PE or recurrent pregnancy loss, should be studied to compare semen induced immune responses. Third, comparisons between new couples versus long-term couples could clarify how repeated partner exposure shapes regulatory responses over time.

## Supplementary Material

hoag064_Supplementary_Data

## Data Availability

The data that support the findings of this study are available from the corresponding author upon reasonable request.
