# Supplementary material for: Heterogeneity in semen-mediated tolerogenic responses: defining immunological determinants of reproductive compatibility
Source: Hum Reprod Open. 2026 Jul 15;2026(3):hoag064. doi: 10.1093/hropen/hoag064 (PMC13446728; doi:10.1093/hropen/hoag064)
Supplement: Supplementary file 1 [file hoag064_supplementary_data.docx]

**Supplementary Tables and Figures**

**Heterogeneity in semen-mediated tolerogenic responses: Defining immunological determinants of reproductive compatibility**

Shahrokh Paktinat^1^, Michael G. Gravett^1^, Sean M. Hughes^1^, Abigail L. P. Spray^1^, Germán G. Gornalusse^1,2^, Florian Hladik^1,2,3^, Lucia Vojtech^1^*

^1^Department of Obstetrics and Gynecology, University of Washington, Seattle, WA, USA

^2^Vaccine and Infectious Disease Division, Fred Hutchinson Cancer Center, Seattle, WA, USA

^3^Department of Medicine, University of Washington, Seattle WA, USA

*Correspondence address:

Department of Obstetrics and Gynecology, University of Washington, 850 Republican St. Box 358055, Seattle, WA 98109, USA

ORCID ID: 0000-0003-2343-0719

email address: [luciav@uw.edu](mailto:luciav@uw.edu)

**Supplementary Table S1.** 15-color flow cytometry panel used for characterization of CD4 T cells and identification of regulatory T cells (Tregs).

**Supplementary Table S2.** 16-color flow cytometry panel used to assess activation-induced marker (AIM) expression on CD4 T cells and regulatory T cells (Tregs).

**Supplementary Figure S1.** Different isoforms of human leukocyte antigen G (HLA-G) are present in semen. Lysates from cell lines and extracellular vesicles (EVs) were used as controls. Approximately 30 micrograms of total protein from each sample were loaded for SDS-PAGE followed by western blotting. Samples included lysates from THP-1 cell line (HLA-G negative control), JEG-3 cell line (HLA-G positive control), EVs from perfused placentas (HLA-G positive vesicle control), and different pools of semen EVs (SEV) and vesicle-depleted semen plasma (VDSP), each consisting of five donors. Red arrow indicates larger HLA-G band present in placental EVs and SEV but not VDSP, likely a membrane-bound isoform of HLA-G.

**Supplementary Figure S2.** Individual semen samples contain variable human leukocyte antigen G (HLA-G) cargo. Lysate from JEG-3 cell line was used as a positive control. Approximately 30 micrograms of total protein or the maximum amount from each sample were loaded for SDS-PAGE followed by western blotting. Red arrows indicate the higher molecular weight (MW) HLA-G band present in semen extracellular vesicles (SEV) but not vesicle-depleted semen plasma (VDSP), likely a membrane-bound isoform of HLA-G. Purified SEV d, e, and f contain little or no HLA-G membrane isoforms. SEC: size-exclusion chromatography.

**Supplementary Figure S3.** Semen extracellular vesicles (SEV) carries CD73. Approximately 30 micrograms of protein from 4 individual vesicle-depleted semen plasma (VDSP) and SEV samples were loaded for SDS-PAGE followed by western blotting.

**Supplementary Figure S4.** Gating strategy for characterizing CD4 T cells after co-culture with semen extracellular vesicles (SEV) and vesicle-depleted semen plasma (VDSP) -exposed monocyte-derived dendritic cells (MoDCs). FMO: Fluorescence Minus One control; CTLA-4: cytotoxic T-lymphocyte-associated protein 4; PD-1: programmed cell death protein 1; TIGIT: T-cell immunoreceptor with Ig and ITIM domains; AREG: amphiregulin; FOXP3: Forkhead box P3.

**Supplementary Figure S5.** Heatmaps depicting increase in each regulatory T cell (Treg) phenotype normalized to the highest increase observed in each cell population, across all participants. SEV: semen extracellular vesicles; VDSP: vesicle-depleted semen plasma; CTLA-4: cytotoxic T-lymphocyte-associated protein 4; PD-1: programmed cell death protein 1; TIGIT: T-cell immunoreceptor with Ig and ITIM domains; AREG: amphiregulin; Tr1: type 1 regulatory T cell.

**Supplementary Figure S6.** Frequencies of FOXP3+ regulatory T cell (Treg) in response to reduced concentrations of vesicle-depleted semen plasma (VDSP). Results from unique recipient cell cultures are shown as individual dots, bars and lines indicate mean and standard deviation. Exposed once: cells harvested at day 7 at the end of the initial monocyte-derived dendritic cells (MoDCs)/naïve CD4 T-cell co-culture, with no further stimulation during day 7-8. Linked re-exposure: day 7 harvested cells re-stimulated for 24 h (day 7-8) with VDSP fractions from the same semen sample used for priming. Unlinked re-exposure: day 7 harvested cells re-stimulated for 24 h (day 7-8) with VDSP fractions from a different semen sample than that used for priming. FOXP3: Forkhead box P3.

**Supplementary Figure S7.** Frequencies of CD137+ CXCR4- regulatory T cell (Treg) in response to reduced concentrations of vesicle-depleted semen plasma (VDSP). Results from unique recipient cell cultures are shown as individual dots, bars and lines indicate mean and standard deviation. Exposed once: cells harvested at day 7 at the end of the initial monocyte-derived dendritic cells (MoDCs)/naïve CD4 T-cell co-culture, with no further stimulation during day 7-8. Linked re-exposure: day 7 harvested cells re-stimulated for 24 h (day 7-8) with VDSP fractions from the same semen sample used for priming. Unlinked re-exposure: day 7 harvested cells re-stimulated for 24 h (day 7-8) with VDSP fractions from a different semen sample than that used for priming.
